# Combining thermocompression, multi-enzymolysis, and citric acid-grafting or carboxymethylation on the physicochemical and hypoglycemic properties of *Ziziphus jujuba* pit dietary **fiber**

**DOI:** 10.3389/fnut.2025.1631421

**Published:** 2025-07-23

**Authors:** Xinling Song, Yong Yang, Chen Feng, Peiyao Long, Yan Li, Biao Ma, Xinyue Meng, Yueyue Niu, Yaqi Li

**Affiliations:** ^1^Food Science College of Shanxi Normal University, Taiyuan, China; ^2^Shanxi Province Cancer Hospital, Taiyuan, China; ^3^Shanxi Hospital Affiliated to Cancer Hospital, Chinese Academy of Medical Sciences, Taiyuan, China; ^4^Cancer Hospital Affiliated to Shanxi Medical University, Taiyuan, China

**Keywords:** jujube pit dietary fiber, thermocompression, citric acid-grafting, multi enzymolysis, carboxymethylation, hydration properties, hypoglycemic properties

## Abstract

**Introduction:**

Red jujube (*Ziziphus jujuba* Mill.) pits are a rich, available, and inexpensive dietary fiber resource. However, red jujube pits are rarely used in the food industry because of their hardness and high content of cellulose and lignin.

**Methods:**

Herein, the influences of combining thermocompression, enzymolysis, citric acid-grafting, or carboxymethylation on the physicochemical and hypoglycemic properties of jujube pit dietary fiber (JPDF) were investigated.

**Results and discussion:**

A lower crystallinity, more porous microstructure, smaller particle size, higher viscosity, expansion volume, and ability to retain water were observed in JPDF after thermocompression and enzymolysis combined with citric acid-grafting or carboxymethylation (*P* < 0.05). JPDF modified via thermocompression, enzymolysis, and carboxymethylation (JPDF-TEC) exhibited the largest surface area (189.21 m^2^·kg^−1^), highest viscosity (16.31 cP), expansion volume (8.70 ml/g), glucose diffusion-inhibitory activity (46.63%), and α-glycosidase-inhibitory activity (20.35%). Moreover, JPDF modified via thermocompression, enzymolysis, and citric acid-grafting (JPDF-TECA) exhibited the highest soluble fiber content (24.00 g/100 g), glucose sorption quantity (34.79 μmol·g^−1^), and α-amylase-inhibitory activity (23.43%). However, thermocompression and enzymolysis, coupled with citric acid-grafting or carboxymethylation, reduced the lightness of JPDF. Thus, thermocompression and enzymolysis, coupled with carboxymethylation, was the most effective method for improving the hypoglycemic properties and applications of JPDF.

## 1 Introduction

Red jujube (*Ziziphus jujuba* Mill.) is widely planted in northern and northwest China, especially in the provinces of Shanxi, Hebei, Henan, and Xinjiang (1). A substantial quantity of jujube pits is generated during the processing of red jujube. These pits are rich in dietary fiber (22.06 g/100 g) and have an annual yield of 150,000 tons (2). Although a small portion is used in medicine, most jujube pits are discarded, mainly ascribed to their high hardness and high content of cellulose and lignin (18.30 g/100 g) (3, 4). Jujube pit dietary fiber (JPDF) has considerable water-expansion volume and viscosity, but its soluble fiber content is low and its adsorption ability and hydration properties are poor (5). To expand the applications of JPDF, it is necessary to increase its soluble fiber content, hydration, and hypoglycemic properties; however, to the best of our knowledge, there are limited data on the modification of JPDF (6). Dietary fiber (DF) plays a crucial role in sugar metabolism balance. Consuming an adequate amount of DF could help prevent hyperglycemia and related diseases (7). Hyperglycemia leads to fat, diabetes, visual deprivation, myocardial infarction, coronary heart disease, stroke, and other cardiovascular diseases (8). Compared to insoluble dietary fiber, soluble dietary fiber has been observed to be better at preventing obesity, hyperglycemia, diabetes, gastrointestinal inflammation, and caner (9, 10). Moreover, the hypoglycemic activities of DFs are dependent on soluble dietary fiber content, microstructure, hydration properties, and functional groups (11, 12). A healthy individual is advised to consume 25–35 g dietary fiber and at least 8 g soluble fiber every day (FAO/WHO) (8). However, DFs from most cereals and vegetables have a low content of soluble dietary fiber (13). The limited number of free hydroxyl groups and the crystal structure are the main factors contributing to the poor hydration properties of DFs, thereby restricting their applications in food products (14). The hydrophilicity of DFs can be dramatically improved by cracking their crystal structure or exposing more hydrophilic groups. Thus, some physical and biological modifications, such as ultrasound, air-excursion, ultra-pressure, thermocompression, and enzymolysis, have been used to break down the crystal structure of DFs and expose more hydrophilic groups (7, 15, 38); meanwhile, chemical methods, such as acetylation, carboxymethylation, and hydroxypropylation, have been employed to improving the hydration and functional properties of DFs by replacing the free hydroxyl groups with acetyl, carboxymethyl, and hydroxypropyl groups, respectively (16). Among these modifications, thermocompression is an efficient method to crack the crystal structure of DFs and expose more hydrophilic groups (17). Cellulase, xylanase, and laccase can cause the degradation of cellulose, hemicellulose, and lignin, respectively, leading to an improvement in hydration and hyperglycemia activities of DFs (17). Carboxymethylation and citric acid-grafting have been proven to be effective methods to improve the hydration and hypoglycemic properties of DFs through grafting more hydrophilic carboxymethyl and citric acid groups on DFs (18, 19); however, there are limited data regarding the synergistic effects of thermocompression and enzymolysis combined with carboxymethylation or citric acid-grafting on hydration and hypoglycemic properties of DFs.

The purpose of the current study was to study the effects of thermocompression and enzymolysis combined with citric acid-grafting or carboxymethylation on the structure, polarity, physicochemical properties, and *in vitro* hypoglycemic activity of JPDF. Another objective was to expand the usage of JPDF in the food industry.

## 2 Materials and methods

### 2.1 Materials

Jujube pits were purchased from the Maishu Red Jujube Processing Factory in Linxian, China. Cellulase (from *Aspergillus niger*, 1.0 × 10^6^ U/g), amyloglucosidase (*Aspergillus niger*, 1.0 × 10^5^ U/g), acarbose, xylanase (from Trichoderma Viride G, 50 U/mg), α-amylase (from *Bacillus licheniformis*, 5 × 10^4^ U/g), laccase (120 U/g), alkaline proteinase (200 U/mg), *p*-nitrophenol-α-D-glucopyranoside (pNPG), and α-glucosidase (from yeast, 50 U/mg) were purchased from Bohui Biological Reagent Factory, Nanning, China. Chloroacetic acid, thiosalicylic acid, and sodium chloride were analytically pure and purchased from Jinjiang Reagent Factory, Tianjin, China.

### 2.2. Extraction of JPDF

Extraction of jujube pit dietary fiber (JPDF) was conducted using the compound enzyme (α-amylase, Alkaline proteinase, and amyloglucosidase) method, citing the same procedures described by Zheng and Li (38).

### 2.3. Thermocompression and multi-enzymolysis of JPDF

Based on the method of Zheng et al. (20), JPDF (60 g) was heated at 130°C and 0.18 MPa using a high-temperature and high-pressure steam sterilizer (LS-100HD, Xinling Instrument & Equipment Co., Zhengzhou, China) for 37 min. The heating pressure and cooling pressure were 0.1 and 0.15 MPa, respectively. Following the method of Xu et al. (18), the treated JPDF was thoroughly dispersed in deionised water (dH_2_O) at a concentration of 5 mg/ml. Next, the dispersion was adjusted to pH 4.8 ± 0.2 using HCl (0.1 mol/L), and then 40 mg of xylanase and cellulase (80 mg) were added. The dispersion was shaken at 3,200 rpm using a Xiao-M vortex oscillator (Xizi Science and Technology Equipment Co., Hangzhou, China) for 6 s, and then stirred at 204 rpm and 50°C for 150 min. After that, the reaction temperature was lowered to 25°C, and then 40 mg of laccase was added. The enzymatic hydrolysis reaction was continued at 204 rpm and 25°C for 65 min. Then, the dispersion in the flask was placed in a 100°C water bath for 10 min, and then cooled and filtered using ARWTman1107-C04 paper. After 6 h of hydration at 55°C using the DHG-9145AE air blast drying oven, thermocompression and multi-enzymolysis-treated jujube pit dietary fiber (JPDF-TE) was obtained.

### 2.4. Carboxymethylation of JPDF-TE

Citing the procedures described by Xu et al. (18), JPDF-TE (3 g) was dispersed in 85% ethanol (*v*/*v*, 120 ml) and stirred at 120 r/min for 30 min. Meanwhile, 5 ml of NaOH (2.13 mol/L) was mixed with 20 ml of 85% ethanol (*v*/*v*) and poured into the JPDF-TE ethanol dispersion. The reaction system was shaken at 3,000 r/min for 12 s, and then heated and alkalised at 53°C in the TS-110X50 water bath thermostatic vibrator (205 r/min) for 1 h. Next, 1.2 ml of NaOH (2.13 mol/L, dissolved in 85% ethanol) and 1 ml of chloroacetic acid (1.17 mol/L, dissolved in 85% ethanol) were added. The reaction dispersion was shaken at 205 r/min and 35°C for 30 min, and then heated at 48°C and 175 r/min for 2.5 h. Then, the dispersion was cooled to 25 ± 1°C, adjusted to pH 7.0 using 50% acetic acid (*v/v*), and sealed in a dialysis membrane (3,500 Da MWCO). After 48 h of dialysis against dH_2_O at 4°C, the dialysate was lyophilised, and JPDF modified via thermocompression and multi-enzymolysis combined with carboxymethylation (JPDF-TEC) was obtained.

### 2.5. Citric acid-grafting of JPDF-TE

First, JPDF-TE was alkalised according to the method by Wu et al. (21) with some modifications. In brief, 5 g of JPDF-TE were dispersed in 250 ml of NaOH (4.5 mol/L) and shaken at 185 rpm and 25 ± 1°C using the TS-110X50 thermostatic vibrator for 180 min. The dispersion was filtered. The residues on the filter paper were washed using dH_2_O until the pH value of the filtrate was 7.0 ± 0.1, and then dried at 60 ± 2°C for 12 h. Next, the dried residues and 0.4 g of Na_2_HPO_4_ were thoroughly dispersed in 100 ml of citric acid aqueous solution (20 mmol/L). The mixture was shaken at 185 rpm and 60 ± 1°C for 45 min. Then, 100 ml of acetone was poured into the reaction dispersions and stirred at 65 rpm. After 30 min, the dispersions were centrifuged at 6,000 *g* for 35 min, and the precipitate was pooled and dried at 60 ± 1°C for 24 h. The dried precipitate was esterified at 135 ± 1°C for 90 min, and JPDF modified via thermocompression, enzymolysis, and citric acid-grafting (JPDF-TECA) was obtained.

### 2.6. Compositional Analysis

The chemical constituents of JPDF, JPDF-TECA, and JPDF-TEC, including protein, moisture, fat, and ash content determination, were obtained following the procedures of AOAC.920.39, AOAC.924.05, AOAC.92.05, and AOAC.955.04, respectively (22). Insoluble fiber contents, including cellulose, hemicellulose, and lignin contents, were calculated based on acid and neutral detergent fibers, and insoluble acid lignin contents were measured using the method by Chu et al. (23). The determination of soluble fiber contents was conducted using the procedures from the AOAC.991.43 method (22), and the total fiber content was the sum of the soluble and insoluble fiber content. Moreover, phenolic content determination was done with the Folin–Ciocalteu method (24).

### 2.7. Color difference and surface area measurement

The color difference (Δ*E*) and surface area of JPDFs were determined using a CD-HNI30 Precision colorimeter (Color Spectrum Technology Co., Ltd, Hangzhou, China) and a Bettersize2600 Laser Particle Size Analyzer (Dandong Baxter Instrument Co., Ltd., Dandong, China), respectively. The Δ*E* between the modified JPDFs and the untreated JPDF was calculated by comparing their lightness (*L*), yellowness (*b*), and redness (*a*) (16).

### 2.8. Structural characteristics

#### 2.8.1 Surface microstructure scanning

The surface microstructure scanning of dry JPDFs coated with gold (10 nm) was carried out on a JOLE-JSM-5070E electron microscope (Japan Electronics Co., Ltd., Beijing, China). Scanning was performed at an accelerating voltage of 10,000 V, and the picture was captured at 2,000 × (measuring scale of 5 μm).

#### 2.8.2. Fourier-transformed infrared spectroscopy

Dry KBr (20 mg) was thoroughly mixed with the JPDFs (1 mg) under an NL-3C Infrared baking lamp, and then pressed into 1–2 mm sheets (17). The sheets were analyzed with a Fourier-transform infrared (FT-IR) spectrometer (LIDA-20, Hengchuanglida Precision Instrument Co., Ltd, Tianjin, China) at a wavenumber range of 4,000–400 cm^−1^.

#### 2.8.3. X-ray diffraction

According to the procedures described by Ji et al. (7), a D-POWER X-ray diffractometer (Guoke Instrument Technology Co., Hefei, China) was employed to scan the crystal structure of JPDFs with a 2θ range of 5–70°. The lowest intensity (*I*_*am*_) and maximum intensity (*I*_002_) were determined, and Equation 1 was used to quantify the crystallinity:


(1) $$\begin{array}{l} Crystallinity\;\left(\%\right)=\left(1-I_{am}/I_{002}\right)\times 100 \end{array}$$

### 2.9. Hydration properties

#### 2.9.1 Ability to retain water

Approximately 1 g of JPDFs (*M*_0_) was mixed with 30 ml of dH_2_O and stirred at 185 rpm and 25 ± 1°C using the TS-110X50 water bath thermostatic vibrator for 120 min (38). After centrifugation at 3,300 × *g* using a TGL-16.5M centrifuge (Luxiangyi Laboratory Instrument Co., Ltd, Shanghai, China) for 15 min, the supernatant was discarded, and the residue in the tube was weighed (*M*_2_). The ability to retain water (ARW) was calculated using Equation 2:


(2) $$\begin{array}{l} ARW\;\left(g/g\right)=\left(M_{1}-M_{0}\right)/M_{0} \end{array}$$

#### 2.9.2 Expansion volume in water

Dry JPDFs (~2 g) were placed in a glass measuring cylinder, and the volume was measured (*V*_0_). Then, 70 ml of dH_2_O was added to the measuring cylinder and left to stand at 25 ± 1°C for 20 h. Then, the volume of the wet JPDFs was measured (*V*_1_). The expansion volume in water (EVW) was calculated using Equation 3:


(3) $$\begin{array}{l} EVW\;\left(g/g\right)=\left(V_{1}-V_{0}\right)/M_{0} \end{array}$$

#### 2.9.3 Viscosity

Following the procedure described by Xu et al. (18), JPDFs dispersion (1 g/25 ml) was measured using an NV-2T digital display rotary viscometer (AMETEK–Brookfield Co. Ltd., Guangzhou, China) at 25 ± 1°C, with a viscosity range of 1–320 cP.

### 2.10. *In vitro* hypoglycemic properties

#### 2.10.1 De-sugarisation

JPDF, JPDF-TECA, and JPDF-TEC (2 g) were dispersed in 85% methanol solution (v/v) (18). The dispersions were shaken at 185 rpm and 55 ± 1°C using the TS-110X50 water bath thermostatic vibrator. After 3 h, the dispersions were filtered with ARWtman1107-C04 paper. The residue was dehydrated at 60 ± 1°C for 4 h to obtain desugared samples.

#### 2.10.2 Glucose sorption quantity

A series of glucose solutions with different concentrations (0.1–0.5 g/L) were thoroughly mixed with 2 g JPDFs at 3,200 rpm using the Xiao-M vortex oscillator for 7 s (25). Next, the dispersions were transferred to the TS-110X50 thermostatic vibrator and shaken at 185 rpm and 37 ± 1°C. After 3 h, the dispersions were filtered using 120-mesh nylon cloth, and the glucose concentration in the filtrate solution was measured using the 3, 5-dinitrosalicylic acid method (26). The reduction in glucose amount of the dispersions per the weight of JPDFs was defined as the glucose sorption quantity (GSQ) of JPDFs.

#### 2.10.3 Glucose diffusion-inhibitory activity

Based on the modified procedures described by Xu et al. (18), 40 ml of glucose aqueous solution (55.51 μmol/L) was thoroughly mixed with JPDFs (1 g). The dispersion was sealed in a JNH-2 dialysis membrane (3,500 Da MWCO, Tengjing Ultra-filter Material Co. Ltd., Nanjing, China). The dialysis was performed against 300 ml of dH_2_O at 185 rpm and 37 ± 1°C using the TS-110X50 thermostatic vibrator for 120 min. The glucose concentration of the dialysate solution was quantified at 20 min intervals using the 3, 5-dinitrosalicylic acid method (26). Diffusion kinetics of glucose in the presence of JPDFs was drawn. The control was done using the same procedures described above without JPDFs. The reduction in glucose diffusion rate relative to the glucose diffusion rate in the control was defined as the glucose diffusion-inhibitory activity (GDIA).

#### 2.10.4 Amylolysis and α-amylase inhibitory activity

Following the modified procedures described by Berktas and Cam (25), 1 mg/ml of soluble starch aqueous solution (40 ml) was thoroughly mixed with 0.15 g JPDFs at 3,200 rpm using the Xiao-M vortex oscillator for 7 s. The dispersion was adjusted to pH 6.8 ± 0.1, and 25 mg of α-amylase was added. The dispersion was sealed in a JNH-2 dialysis membrane (3,500 Da MWCO). Next, the dispersion in the membrane was dialyzed against 300 ml of dH_2_O at 185 rpm and 37 ± 1°C using the TS-110X50 thermostatic vibrator for 120 min. The same system without JPDFs was used as a control. The concentration of sugar dialyzed out was determined at 20 min intervals using the 3, 5-dinitrosalicylic acid method (26), and amylolysis was drawn. The α-amylase inhibitory activity (α-AIA) was calculated using Equation 4:


(4) $$\begin{array}{l} \alpha -AIA\;\left(\%\right)=\left(P_{C}-P_{S}\right)/P_{C}\times 100\% \end{array}$$

where *P*_*S*_ and *P*_*C*_ are the glucose production amounts in the sample and control groups, respectively.

#### 2.10.5 Influence mechanism of JPDFs on α-amylase by fluorescence

A total of 100 mg of JPDFs was dispersed in 0.1 mol/L of phosphate buffer (1 ml, pH 6.7), and then thoroughly mixed with 150 μg/ml of α*-*amylase solution (9 ml) at 3,200 rpm using the Xiao-M vortex oscillator for 5 s (27). Next, the mixture was oscillated at 65 rpm and 25 ± 1°C using the TS-110X50 thermostatic vibrator for 0.5 h. The fluorescence of the mixture was scanned using a PTI-65 spectrophotometer (Precision Technology Instrument Co., Shanghai, China). The recorded range, excitation wavelength, emission wavelength, and slit width were 290–600, 280, 295, and 5 nm, respectively.

#### 2.10.6 α-glycosidase inhibitory activity

Following the method by Zhong et al. (28), 1 ml of JPDFs dispersion (0.1 mg/ml, in 100 μmol/L of phosphate buffer, pH 6.7) and 0.1 UN of α-glycosidase (2 ml) were vortexed at 3,200 rpm for 6 s. After 10 min of cultivation at 37°C, the dispersion was mixed with 625 μmol/L of *p*-NPG (0.5 ml), and then stirred at 65 rpm and 37°C using the TS-110X50 thermostatic vibrator for 0.5 h. T0.1 mol/L of Na_2_CO_3_ (2.5 ml) was added to terminate the reaction. After 10 min of centrifugation at 3,200*g*, a LJ-UV90 ultraviolet-visible spectrophotometer (Lanjing Electronic Technology Co., Weifang, China) was used to measure the absorbance of the supernatant at 400 nm. The control group was performed using the same procedures without JPDFs, and acarbose (1.5 mmol/L) was used for positive comparison. The α-glycosidase inhibitory activity (α-GIA) was calculated using Equation 5:


(5) $$\begin{array}{l} \alpha -GIA\;\left(\%\right)=\left(A_{C}-A_{S}\right)/A_{C}\times 100 \end{array}$$

where *A*_*C*_ and *A*_*S*_ are the absorbance at 400 nm of the control and sample groups, respectively.

### 2.11. Statistics analysis

Tests were conducted in triplicate, and the statistical analysis (mean ± SD) was conducted using V.17.4 SPSS software (International Business Machines Corporation, Chicago, USA). *Post-hoc* analysis was conducted using Duncan's multiple comparisons, and the threshold for significant difference (*P* < 0.05) was 95%.

## 3 Results and discussion

### 3.1. Synergistic effects on chemical composition of JPDFs

As shown in Table 1, the total dietary fiber content of jujube pit was 22.06 g/100 g, and the insoluble fiber content was 17.76 g/100 g, which was consistent with the results of Agrawal et al. (2). After thermocompression and multi-enzymolysis combined with citric acid-grafting or carboxymethylation, the substitution degrees of JPDF-TEC and JPDF-TECA were 3.52 and 1.19%, respectively. The soluble dietary fiber (SDF) contents of JPDF-TEC and JPDF-TECA were higher than that of JPDF, and their insoluble fiber content was lower than that of JPDF (*P* < 0.05). Thermocompression damaged the crystal structure of DFs and broke the polysaccharide chains; while multi-enzymolysis (cellulase, xylanase, and laccase) degraded the glucosidic bonds, leading to increase in polar groups (20). The grafting of carboxymethyl and citric acid groups has been proven to improve the polarity of DFs (16, 19). JPDF-TEC showed a higher total fiber content than that of JPDF-TECA, but the difference is not significant (*P* > 0.05). During carboxymethylation, the ash, protein, and fat contents of JPDF were decreased, and the total fiber content was increased. Furthermore, JPDF-TECA offered a higher SDF content than JPDF-TEC (*P* < 0.05), depicting that thermocompression and multi-enzymolysis, coupled with carboxymethylation, was less effective for increasing the polarity of JPDF than combining thermocompression, multi-enzymolysis, and citric acid-grafting. The hydrophilicity of citric acid groups was higher than that of carboxymethyl groups (21).

**Table 1:** Proximate compositions of jujube pit dietary fiber (JPDF), JPDF modified by thermocompression, enzymolysis, and citric acid-grafting (JPDF-TECA), and JPDF treated by thermocompression, enzymolysis, and carboxymethylation (JPDF-TEC).

**Constituent**	**Jujube pit powder**	**JPDF**	**JPDF-TEC**	**JPDF-TECA**
Protein (g·100 g^−1^)	2.84 ± 0.05^a^	0.74 ± 0.03^b^	0.33 ± 0.03^c^	0.45 ± 0.09^c^
Fat (g·100 g^−1^)	4.65 ± 0.17^a^	2.84 ± 0.18^b^	1.09± 0.04^c^	1.12 ± 0.06^c^
Moisture (g·100 g^−1^)	5.74 ± 0.33^a^	5.42 ± 0.34^a^	5.04 ± 0.22^a^	6.34 ± 0.33^a^
Ash (g·100 g^−1^)	0.79 ± 0.08^b^	1.02 ± 0.06^b^	1.75 ± 0.09^a^	1.33 ± 0.03^ab^
Total fiber (g·100 g^−1^)	22.06 ± 1.03^b^	88.37 ± 4.19^a^	91.09 ± 4.33^a^	90.35 ± 2.85^a^
Insoluble fiber (g·100 g^−1^)	17.76 ± 0.57^d^	82.43 ± 1.38^a^	71.28 ± 2.48^b^	66.35 ± 2.57^c^
Soluble fiber (g·100 g^−1^)	4.30 ± 0.18^c^	5.94 ± 0.09^c^	18.91 ± 0.18^b^	24.00 ± 1.22^a^
Extractable polyphenols (mg· g^−1^)	3.75 ± 0.16^c^	6.72 ± 0.44^b^	11.78 ± 0.39^a^	10.00 ± 0.56^a^
Cellulose (g·100 g^−1^)	8.33 ± 1.64^c^	41.31 ± 2.51^a^	29.56 ± 1.49^b^	27.67 ± 0.33^b^
Hemicellulose (g·100 g^−1^)	4.95 ± 0.11^c^	24.37 ± 1.02^a^	16.99 ± 0.64^b^	15.94 ± 0.18^b^
Lignin (g·100 g^−1^)	4.97 ± 0.25^d^	25.85 ± 1.90^a^	17.67 ± 0.09^b^	14.22 ± 0.75^c^

Different small letters (a–d) in the same line meant significant difference (*P* < 0.05); all test was replicated at least three times (*n* ≥ 3).

By contrast, the insoluble dietary fiber contents of JPDF, including cellulose, hemicellulose, and lignin contents, were dramatically decreased (*P* < 0.05; Table 1). Cellulase caused the degradation of cellulose and increased the polarity of JPDF (38). Xylanase cracked the glycosidic bonds of hemicellulose, while laccase catalyzed the hydrolysis of lignin and increased the hydrophilicity of JPDF (25). Apart from that, thermocompression and alkalisation processing during carboxymethylation and citric acid-grafting can damage the crystal structure of DFs and reduce cellulose, hemicellulose, and lignin contents (15). Moreover, these synergistic modifications reduced the protein and fat contents of JPDF but increased its ash and extractable polyphenol contents (*P* < 0.05). During thermocompression, multi-enzymolysis, and the alkalisation, the structure of JPDF was expanded and the chemical bonds linking to the polyphenols were broken, resulting in a loss of protein and fat and an increment in extractable polyphenol content (38). Additionally, laccase caused degradation of lignin (polyphenol polymers) and increased extractable phenols consequently (29). Meanwhile, the usage of chemicals during these modifications increased the ash content (30). Ji et al. (7) obtained similar results.

### 3.2. Color and size of JPDFs

The results in Table 2 showed that JPDF-TEC and JPDF-TECA exhibited visible color difference (with higher *a* and *b* values and lower *L* value) compared to JPDF (*P* < 0.05). As *L, a*, and *b* values represented lightness, redness, and yellowness, respectively, the color of JPDF darkened after thermocompression and multi-enzymolysis combined with carboxymethylation or citric acid-grafting. During carboxymethylation, the thermocompression, heating, and alkalisation treatments (2.13 mol/L of NaOH) resulted in the brown reaction and decreased the lightness of JPDF (17), resulting in the highest Δ*E* (19.20) and lowest *L* value. Alternatively, the processing conditions of citric-acid grafting were relatively moderate (19); therefore, JPDF-TECA showed a lower Δ*E* and higher *L* value than JPDF-TEC (*P* < 0.05).

**Table 2:** Color, surface area, and functional properties of JPDF, JPDF-TECA, and JPDF-TECA.

**Properties**	**JPDF**	**JPDF-TECA**	**JPDF-TEC**
a^*^	16.96 ± 0.75^b^	17.78 ± 0.29^a^	18.51 ± 0.45^a^
b^*^	25.55 ± 1.68^b^	29.01 ± 1.52^a^	27.94± 1.78^ab^
L^*^	60.05 ± 4.82^a^	45.09 ± 1.61^b^	41.06 ± 2.75^c^
*ΔE*	Control	15.37^b^	19.20^a^
Surface area (m^2^·kg^−1^)	53.36 ± 4.02^c^	148.93 ± 3.39^b^	189.21 ± 9.21^a^
D_3, 2_ (μm)	137.86 ± 5.88^a^	70.04 ± 0.98^b^	62.97 ± 6.64^c^
Ability to retain water (g·g^−1^)	4.62 ± 0.25^c^	9.92 ± 0.49^a^	8.08 ± 0.39^b^
Expansion volume in water (ml·g^−1^)	4.36 ± 0.20^c^	6.80 ± 0.10^b^	8.70 ± 0.20^a^
Viscosity (cP)	8.73 ± 0.14^c^	14.04 ± 0.08^b^	16.31 ± 1.21^a^
Glucose sorption quantity (μmol·g^−1^)	20.29 ± 1.26^c^	34.79 ± 0.81^a^	25.39 ± 1.08^b^
Glucose diffusion inhibitory activity (%)	23.39 ± 1.12^c^	31.70 ± 2.84^b^	46.63 ± 1.57^a^
α-Glycosidase inhibiting activity (%)	9.98 ± 0.31^c^	14.87 ± 0.58^b^	20.35 ± 1.31^a^
α-Amylase inhibiting activity (%)	10.12 ± 0.73^c^	23.43 ± 1.33^a^	16.54 ± 1.21^b^

Different small letters (a–c) in the same line meant significant difference (*P* < 0.05); all test was replicated at least three times (*n*≥ 3).

The D_3, 2_ values of JPDF-TEC and JPDF-TECA were lower than those of JPDF, and their surface area was larger, which was a result of the degradation of glycosidic bonds caused by thermocompression, multi-enzymolysis, and alkalisation (25, 30). Similar results were obtained by Zheng et al. (20). JPDF-TEC had a larger surface area (189.21 m^2^·kg^−1^) than that of JPDF-TECA (*P* < 0.05), because the time of heating and alkalisation during carboxymethylation was longer than that during citric acid-grafting.

### 3.3 Structural characteristics

#### 3.3.1 Scanning electron microscopy

**Figure 1:**
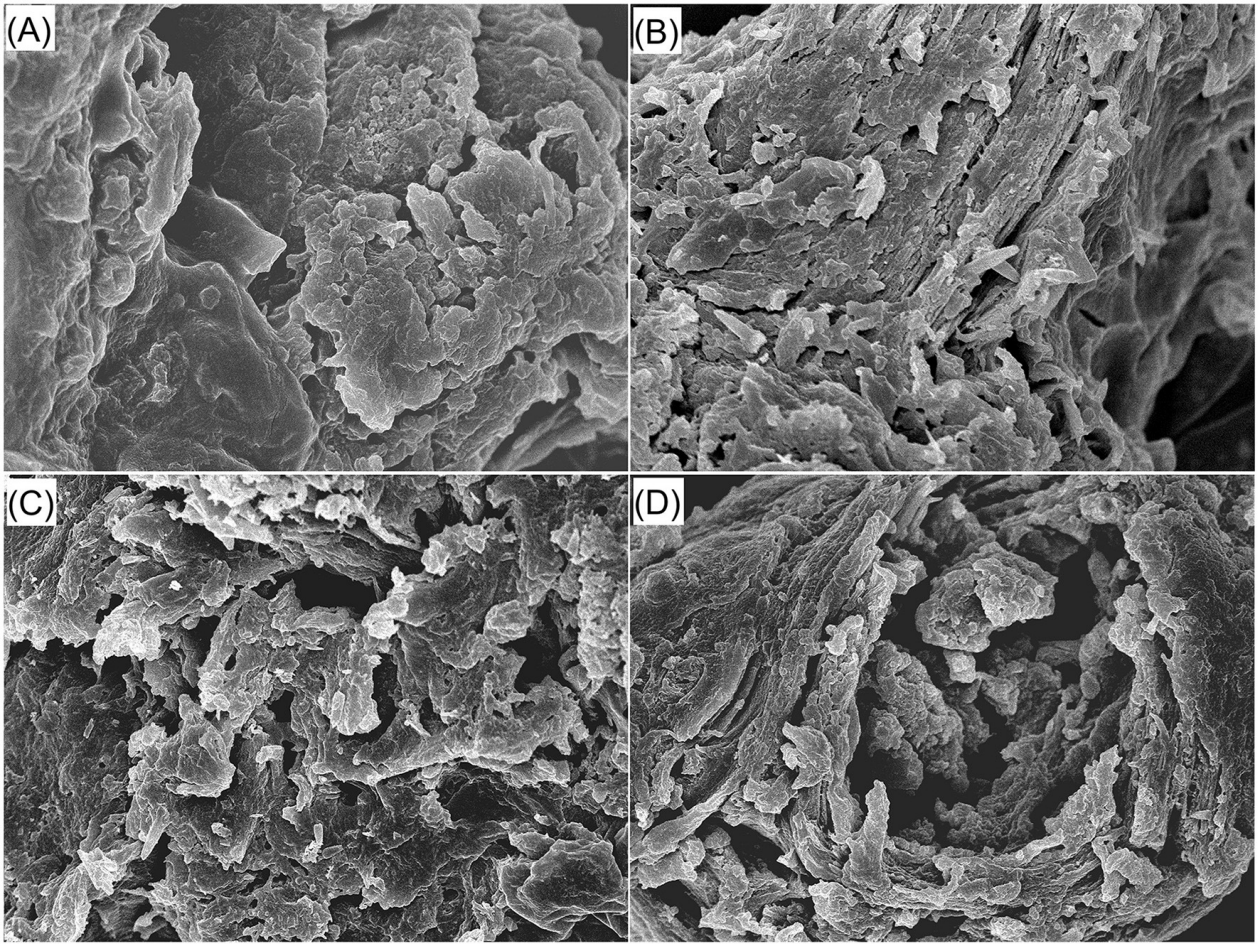
Scanning electron micrographs of red jujube pit powder **(A)**, JPDF **(B)**, JPDF-TEC **(C)**, and JPDF-TEC **(D)** with a magnification of 5,000 × , at 1 μm. JPDF, jujube pit dietary fiber; JPDF-TEC, JPDF treated by thermocompression, enzymolysis and carboxymethylation; and JPDF-TECA, JPDF modified by thermocompression, multi-enzymolysis and citric acid-grafting.

Compared to the microstructure of jujube pits (Figure 1A), there are more cracks and holes in the microstructure of JPDF (Figure 1B). This is mainly ascribed to the loss of proteins, fat, and soluble carbohydrates (Table 1). Furthermore, there were more pores and fragments in the scanning pictures of JPDF-TEC and JPDF-TECA (Figures 1C, D) in comparison to those of JPDF (Figure 1B), which was due to the degradation of glycosidic bonds and reductions in lignin, hemicellulose, and cellulose caused by thermocompression and multi-enzymolysis combined with carboxymethylation or citric acid-grafting (25). Zheng and Li (38) and Xu et al. (18) obtained similar results. The increase in the number of holes and debris was consistent with the decrease in particle size of JPDF-TEC and JPDF-TECA (Table 2), which was conducive for the hypoglycemic effect of DFs (30).

#### 3.3.2 FT-IR

**Figure 2:**
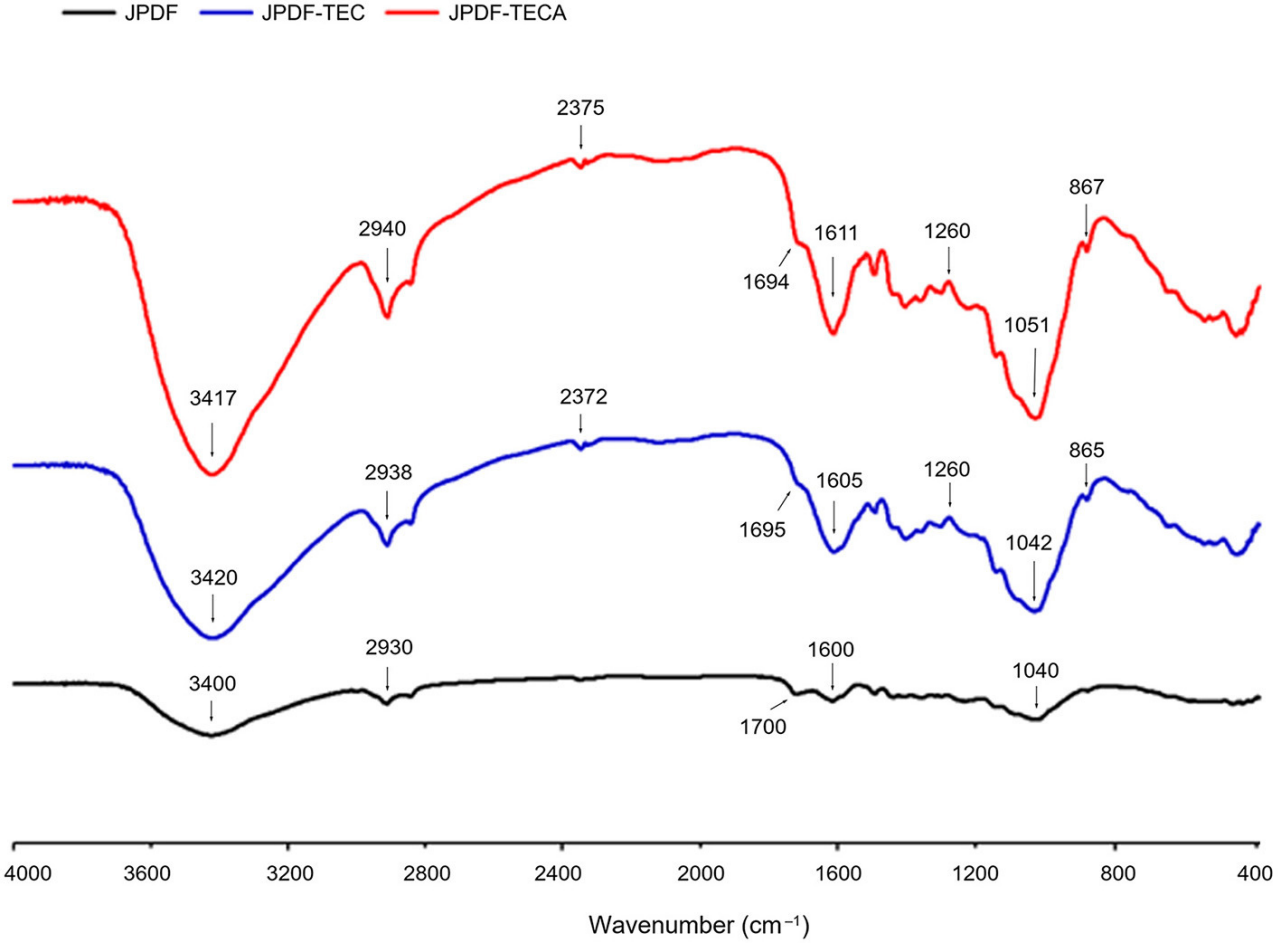
Fourier-transformed infrared spectroscopy of JPDF, JPDF-TEC, and JPDF-TEC.

As shown in Figure 2, the peaks appeared at 3,400, 2,930, 1,700, and 865 cm^−1^ were representative of the hydroxyl (O–H) bonds, carbonyl (C=O), carbon–oxygen (C–O), and carbon–carbon (C–C), respectively (16, 17). A slight difference was observed between the FT-IR spectra of JPDF, JPDF-TEC, and JPDF-TECA. After thermocompression and multi-enzymolysis separately combined with carboxymethylation and citric acid-grafting, the branded peak at 3,400 cm^−1^ in the spectrum of JPDF shifted to 3,420 and 3,417 cm^−1^, respectively, and this phenomenon is ascribed to asymmetric stretching of hydrogen bonds (10). The new peaks that appeared at 867 and 865 cm^−1^ in the spectra of JPDF-TEC and JPDF-TECA were a consequence of the vibration of β-C–H and revealed that β-glycosidic bonds were cracked by thermocompression and multi-enzymolysis (17). The blue shift (from 1,040 to 1,042 cm^−1^) and red shift (from 1,700 to 1,695 cm^−1^) in the spectrum of JPDF-TEC revealed the deformation of the methylene and carboxyl groups, respectively, verifying the grafting of carboxymethyl groups (16). Furthermore, the grating of carbonyl and carboxyl groups in JPDF was revealed via the blue shift (from 2,930 to 2,940 cm^−1^) and the new peaks at 1,260 and 2,370 cm^−1^ in the spectrum of JPDF-TECA (25), proving that citric acid groups were linked to JPDF. These findings demonstrated that the chemical groups and linking bonds of JPDF were altered by those synergistic modifications.

#### 3.3.3 X-ray diffraction

**Figure 3:**
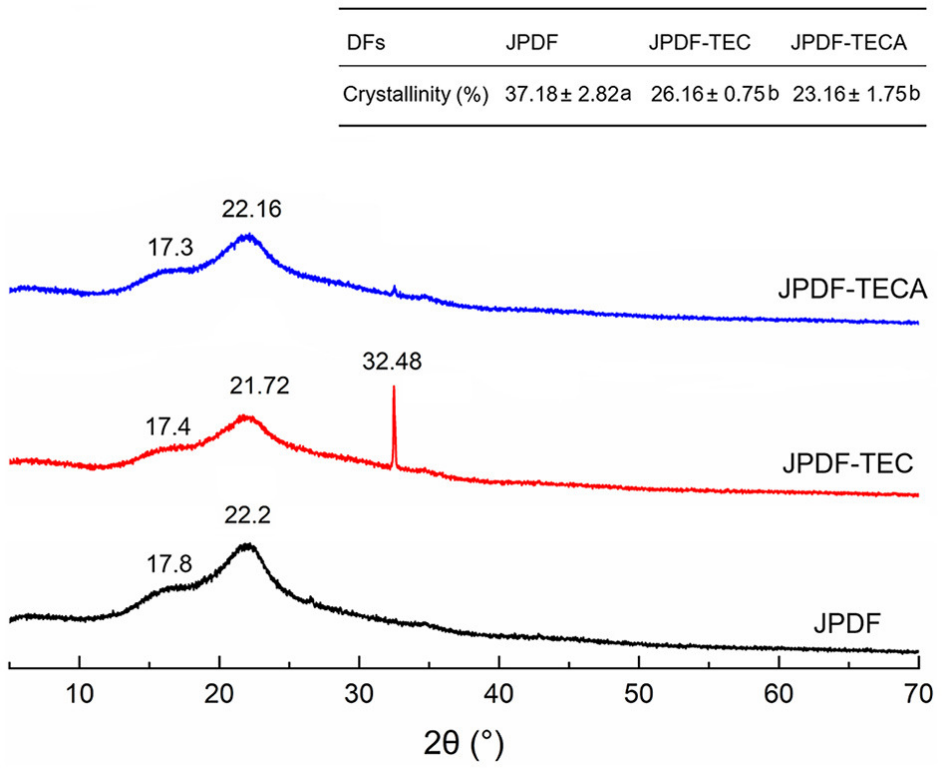
*X*-ray diffraction spectra of JPDF, JPDF-TEC, and JPDF-TEC, and their crystallinity. Different small letters (a and b) in the table indicate significant difference (*P* < 0.05).

X-ray diffraction (XRD) is an efficient technology to investigate the crystal structure of DFs. The XRD spectra and crystallinity of JPDF, JPDF-TEC, and JPDF-TECA are shown in Figure 3. The two brand intensity peaks at 2θ of 22.2 and 17.8 represent the crystal structures I and II, respectively (17). Compared to the spectrum of JPDF, the maximum intensity (*I*_002_) at 2θ of 22.2 transferred to 2θ of 21.72 and 22.16 in the spectra of JPDF-TEC and JPDF-TECA, respectively; while the second maximum intensity at 2θ of 17.8 transferred to 2θ of 17.4 and 17.3, suggesting that the crystal structures I and II of JPDF were damaged by thermocompression and multi-enzymolysis coupled with carboxymethylation or citric acid-grafting (28). Moreover, a new intensity peak appeared at 2θ of 32.48 (belonging to the crystal structure in the form of type I cellulose) in the spectrum of JPDF-TEC (31), indicating that thermocompression and multi-enzymolysis, coupled with carboxymethylation, altered the crystal structure of JPDF. Additionally, the crystallinity of JPDF-TEC and JPDF-TECA was lower than that of JPDF (*P* < 0.05), confirming that these synergistic modifications broke the crystal structure of JPDF, which was an advantage for the interactions of JPDF with water or sugars.

### 3.4 Viscosity, ARW, and EVW of JPDFs

DFs with high EVW and viscosity are effective at enhancing fullness feeling and inhibiting the diffusion and adsorption of sugar and oil (8). As shown in Table 2, the viscosity, ARW, and EVW of JPDF-TEC and JPDF-TECA were higher than those of JPDF (*P* < 0.05); this is ascribed to the higher SDF content (Table 1), larger surface area (Table 2), and the grafting of carboxymethyl and citric acid groups. Moreover, the lower crystallinity (Figure 3), higher porosity quantification (Figures 1C, D), and decrease in D_3, 2_ (Table 2) contributed to the high viscosity, ARW, and EVW of JPDF-TEC and JPDF-TECA. Soluble fibers can improve the affinity of DFs to water, resulting in higher hydrophilicity and viscosity (7). A decrease in particle size will expand the effective contact area between DFs and water, and the holes and debris (Figures 1B, C) are helpful for DFs to capture and retain water. A decrease in crystallinity indicated that the ordered crystal structure of JPDF was disrupted, and many polar groups, such as hydroxyl groups, are exposed, resulting in a stronger affinity to water (10). Additionally, carboxymethyl and citric acid groups increased the steric hindrance between fiber chains and expanded the EVW of JPDF (21). Thermocompression broke the linkages between polysaccharide chains and made the structure of DFs fluffier, thereby increasing the EVW of JPDF (20). Citric acid groups were the main reason for the highest ARW of JPDF-TECA (9.92 g/g).

Compared to JPDF-TECA and JPDF, a higher viscosity (16.31 cP) and greater EVW (8.70 ml/g) were observed in JPDF-TEC (*P* < 0.05), which was attributed to the largest surface area (Table 2) and highest soluble fiber content of JPDF-TEC (24.00 g/100 g). Carboxymethyl groups introduced can enhance the steric hindrance between polysaccharide chains, thereby making JPDF-TEC fluffier in aqueous solution (32). Zhang and Ye (5) modified jujube kernel fiber with carboxymethylation and the product showed a lower viscosity and EVW than those of JPDF-TEC and JPDF-TECA, confirming that thermocompression and multi-enzymolysis combined with carboxymethylation or citric acid-grafting were more effective at enhancing the hydration properties of JPDF.

### 3.5 *In vitro* hypoglycemic properties

#### 3.5.1 Glucose sorption quantity

**Figure 4:**
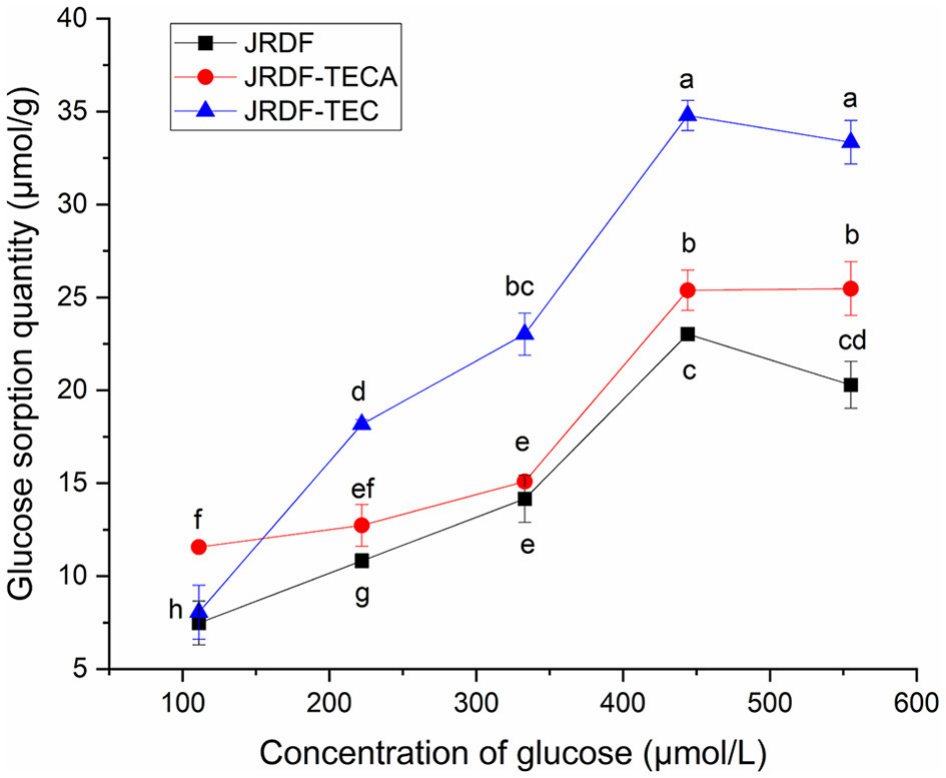
Glucose sorption quantity of JPDF, JPDF-TEC, and JPDF-TEC. Different small letters (a–h) on the data points indicate significant difference (*P* < 0.05).

The isothermal adsorption kinetics and GSQ of JPDFs at different glucose concentrations are shown in Figure 4 and Table 2, respectively. JPDFs exhibited the highest glucose sorption when the concentration of glucose was 444.04 μmol/L. Compared to JPDF, a higher GSQ was observed in JPDF-TEC and JPDF-TECA (*P* < 0.05). After thermocompression and multi-enzymolysis coupled with carboxymethylation or citric acid-grafting, the surface area of JPDF was increased and its contact chance with glucose was increased (9). JPDF-TEC and JPDF-TECA showed porous and fragmented microstructure with many tiny cracks (Figures 1C, D), which was helpful to the adsorption of glucose on DFs (10). Moreover, the grafting of carboxymethyl and citric acid groups remarkably increased the polarity of JPDF, increasing its chemical affinity to glucose. JPDF-TECA exhibited a higher GSQ (34.79 μmol·g^−1^) than JPDF-TEC (*P* < 0.05), because the hydrophilicity of carboxyl groups was higher than that of carboxymethyl groups (30). These results indicated that JPDF-TEC and JPDF-TECA adsorbed glucose mainly through chemical combination. Zheng et al. (20) and Xu et al. (18) found that *Setaria italica* fiber and palm fiber adsorbed glucose mainly by chemical binding.

#### 3.5.2 Delay glucose diffusion

**Figure 5:**
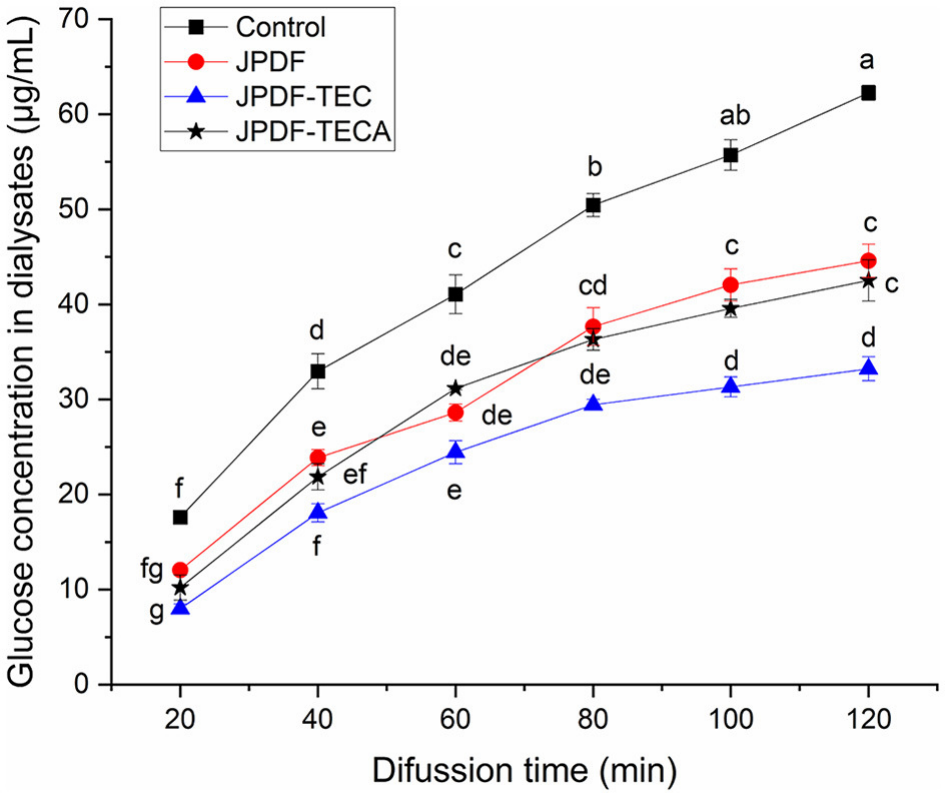
Influences of JPDF, JPDF-TEC, and JPDF-TEC on glucose diffusion. Different small letters (a–g) on the data points indicate significant difference (*P* < 0.05).

Slowing down the diffusion of glucose can decrease the glycemic rising rate (33). The influences of JPDFs on the glucose diffusion kinetics and their GDIA are shown in Figure 5 and Table 2, respectively. JPDF, JPDF-TEC, and JPDF-TECA remarkably slowed down the diffusion rate of glucose. The GDIA of JPDF-TEC and JPDF-TECA was higher than that of JPDF, revealing that thermocompression and multi-enzymolysis combined with carboxymethylation or citric acid-grafting effectively enhanced the delaying effect of JPDF on glucose diffusion. As shown in Tables 1, 2 and Figure 1, JPDF-TEC and JPDF-TECA had higher viscosity, greater EVW, larger surface area, and microstructure with more cracks than JPDF (*P* < 0.05). An improvement in EVW and viscosity was helpful to the inhibition capacity of JPDF-TEC and JPDF-TECA on glucose diffusion (9). The cracks in the microstructure and a larger surface area were an advantage for the adsorption of glucose on DFs (10), resulting in a higher GDIA. In addition, the better GSQ of JPDF-TEC and JPDF-TECA (Figure 4) contributed to their higher GDIA. The higher content of extractable polyphenols (Table 1) facilitated JPDF-TEC and JPDF-TECA to delay glucose diffusion, too (18). Furthermore, the highest GDIA of JPDF-TEC was predominantly attributed to its highest viscosity and the largest EVW and surface area (Table 2). Cellulase hydrolysis, carboxymethylation, and citric acid-grafting have been proven to improve the GDIA of coconut, *Setaria italica*, and basalt dietary fibers, respectively (18, 21). However, the statistical correlation between the GDIA and the viscosity, EVW, and GSQ of JPDFs should be analyzed in the next study.

#### 3.5.3 α-glycosidase inhibiting activity

Inhibiting α-glycosidase can effectively reduce the rate of blood glucose increase (28). JPDF, JPDF-TEC, and JPDF-TECA exhibited a lower α-glycosidase inhibiting activity than that of Acarbose (a α-glycosidase inhibitor that is widely used in the treatment of diabetes) (8). A higher α-GIA was observed in JPDF-TEC and JPDF-TECA compared to that of JPDF (*P* < 0.05). The higher viscosity and EVW made JPDF-TEC and JPDF-TECA (Table 2) more effective at preventing interaction between α-glycosidase and its substrate (*p*-NPG). Carboxymethyl and citric acid groups increased the polarity of JPDF-TEC and JPDF-TECA, further enhancing the interactions between α-glycosidase and JPDF-TEC and JPDF-TECA (12). As polyphenols are efficient inhibitors of α-glycosidase (18), the higher content of extractable polyphenols (Table 1) was one reason for the higher α-GIA of JPDF-TEC and JPDF-TECA. Furthermore, JPDF-TEC showed a higher α-GIA (20.35%) than JPDF-TECA (*P* < 0.05), which was probably ascribed to its higher soluble fiber content (Table 1) and the stronger affinity between α-glycosidase and the carboxymethyl groups of JPDF-TEC. Carboxymethylation has also been shown to significantly improve the α-GIA of coconut cake fiber (20).

#### 3.5.4 Effects on α-amylase activity and starch enzymolysis kinetics

**Figure 6:**
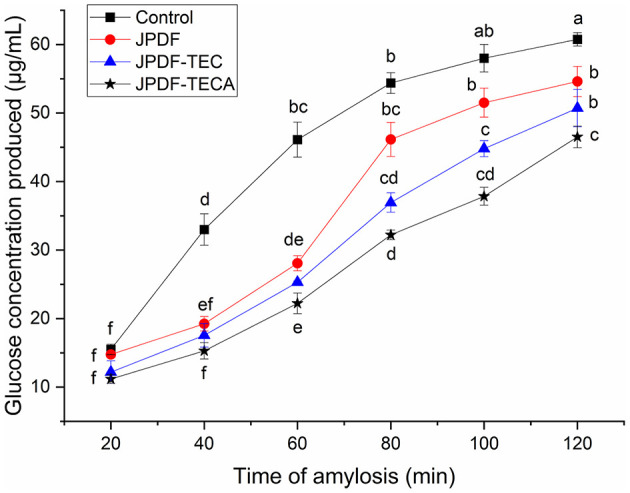
Influences of JPDF, JPDF-TEC, and JPDF-TEC on amylolysis kinetics. Different small letters (a–f) on the data points indicate significant difference (*P* < 0.05).

α-Amylase catalyzes the hydrolysis of starch, which is crucial for maintaining stable glycemic levels. Inhibiting the hydrolysis of starch can effectively alleviate hyperglycemia (28). The effects of JPDFs on the starch enzymolysis kinetics are shown in Figure 6, and their α-AIA are shown in Table 2. JPDF, JPDF-TEC, and JPDF-TECA effectively restrained the hydrolysis of starch and showed considerable α-AIA (10.12%−23.43%). The JPDFs inhibited α-amylase via physically restraining the contact chance between starch and α-amylase, altering the structure of α-amylase, or changing the catalysis center's polar microenvironment (7). JPDF-TECA and JPDF-TEC exhibited a higher α-AIA than JPDF (*P* < 0.05), mainly ascribed to their porous microstructure with more cracks (Figures 1C, D), greater surface area, higher viscosity and EVW (Table 2), and better abilities to adsorb glucose and delay glucose diffusion (Figures 4, 5). A more porous microstructure, larger surface area, and better ability to adsorb glucose were advantage for JPDF-TECA and JPDF-TEC to physically restrain the touching chance between starch and α-amylase (27); while a better ability to delay glucose diffusion indicated that DFs have a product stacking effect (25). The higher extractable polyphenol content (Table 1) was another reason for the better α-AIA of JPDF-TECA and JPDF-TEC, because polyphenols have been proven to inhibit α-amylase. Zhong et al. (28) found that an increment in the hydration properties and glucose adsorption capacity improves the α-AIA of DFs. Alternatively, the α-AIA of JPDF-TECA (23.43%) was higher than those of carboxymethylated *Setaria italica* fiber (13.39%) (18) and cellulase and heating-treated coconut fiber (11.35%) (20), highlighting that thermocompression and enzymolysis combined with citric acid-grafting was a better choice to restrain α-amylase. Although JPDF-TEC exhibited higher viscosity, EVW, GDIA, and surface area (Table 2), it showed a lower α-AIA than that of JPDF-TECA (*P* < 0.05), because the carboxyl groups in JPDF-TECA had stronger interactions with α-amylase than the carboxymethyl groups in JPDF-TEC (30).

#### 3.5.5 Effects on the conformation of α-amylase

**Figure 7:**
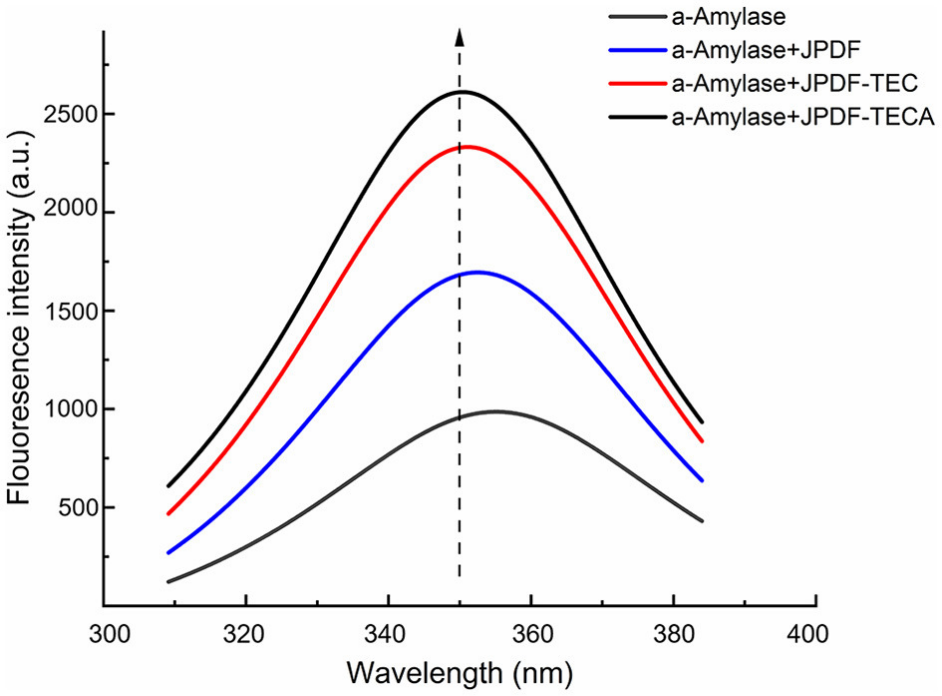
Quenching influences of JPDF, JPDF-TEC, and JPDF-TEC on the fluorescence of α-amylase.

Fluorescence spectroscopy scanning was used to investigate the special effect mechanism of the JPDFs on α-amylase. As shown in Figure 7, an obvious quenching effect was observed on the fluorescence intensity of α-amylase after the addition of JPDF, JPDF-TEC, and JPDF-TECA (Figure 7), corresponding to their α-amylase inhibiting activity (Table 2). Compared to JPDF, JPDF-TEC and JPDF-TECA were more effective in quenching the fluorescence of α-amylase, demonstrating that thermocompression and multi-enzymolysis coupled with citric acid-grafting or carboxymethylation improved the effects of JPDF on the conformation of α-amylase (34). The fluorescence peak of α-amylase that appeared at (~350 nm transferred to 353, 354, and 358 nm after the addition of JPDF, JPDF-TEC, and JPDF-TECA, respectively, indicating that these JPDFs can bind to the residues of α-amylase or can change its catalysis center's polar microenvironment (7, 35). The influence of JPDFs on α-amylase's conformation was the main reason for their considerable α-AIA (Table 2) (36). JPDF-TECA exhibited the most efficient quenching effect on the fluorescence of α-amylase, which was responsible for its highest α-AIA (37).

In general, the results shown in Figures 4–7 and Table 2 reveal that JPDF-TEC, JPDF-TECA, and JPDF can exhibit a hypoglycemic effect via adsorbing glucose, delaying the diffusion of glucose, inhibiting α-glycosidase, or restraining α-amylase. Furthermore, thermocompression and multi-enzymolysis combined with carboxymethylation or citric acid-grafting significantly improved the hypoglycemic activities of JPDF.

## 4 Conclusion

Lowering the impact on the crystal structure and improving effects in the soluble fiber content (from 5.94 to 24.00 g·100 g^−1^), microstructure, hydration properties, and hypoglycemic activity of JPDF were observed on thermocompression and multi-enzymolysis combined with citric acid-grafting or carboxymethylation. Thermocompression, multi-enzymolysis, and carboxymethylation was the most efficient method to increase the surface area, viscosity (from 8.73 to 16.31 cP), EVW (from 4.36 to 8.70 ml·g^−1^), and α-GIA (from 9.98 to 20.35%) of JPDF; while thermocompression and multi-enzymolysis combined with acid-grafting was better at improving the solubility (24.00 g/100 g), GSQ (34.79 μmol·g^−1^), and α-AIA (23.43%) of JPDF. Therefore, thermocompression and enzymolysis combined with carboxymethylation was the most effective method to improve the hydration and hypoglycemic properties of JPDF. However, the statistical correlation between the hydration properties and hypoglycemic activities of JPDF should be studied in future research. Additionally, the specific mechanism by which thermocompression and enzymolysis, coupled with carboxymethylation or citric acid-grafting, influence the *in vivo* hypoglycemic effect of JPDF need further investigation.

## Data Availability

The original contributions presented in the study are included in the article/supplementary material, further inquiries can be directed to the corresponding author.

## References

[1] Li M Geng Z Zhang X Yang X Zhang Q. Establishment and application of a simulation model for infrared radiation combined with hot air intermittent drying of jujube slices. LWT-Food Sci Tech. (2025) 215:117280. 10.1016/j.lwt.2024.117280

[2] Agrawal P Singh T Pathak D Chopra H. An updated review of *Ziziphus jujube:* major focus on its phytochemicals and pharmacological properties. Pharmacol Res-Mod Chin Med. (2023) 8:100297. 10.1016/j.prmcm.2023.100297

[3] Maheri H Hashemzadeh F Shakibapour N Kamelniya E Malaekeh-Nikouei B Mokaberi P. Glucokinase activity enhancement by cellulose nanocrystals isolated from jujube seed: a novel perspective for type II diabetes mellitus treatment (*in vitro*). J Mol Stru. (2022) 1269:133803. 10.1016/j.molstruc.2022.133803

[4] Wang N Li Q Liu M Liu M Zhao Z. Structural characterization of alkali-extracted jujube polysaccharides and their effects on the fecal microbiota *in vitro*. LWT-Food Sci Technol. (2023) 184:115087. 10.1016/j.lwt.2023.115087

[5] Zhang J Ye Z. Influences of superfine-grinding and mix enzymatic hydrolysis combined with hydroxypropylation or acetylation on the structure and physicochemical properties of jujube kernel fiber. Front Sustain Food Syst. (2024) 8:1382314. 10.3389/fsufs.2024.1382314

[6] Al-Khalili M Al-Habsi N Al-Alawi A Al-Subhi L Myint MTZ Al-Abri M. Structural characteristics of alkaline treated fibers from date-pits: residual and precipitated fibers at different pH. Bioact Carbohydr Diet Fibre. (2021) 25:100251. 10.1016/j.bcdf.2020.100251

[7] Ji R Zhang X Liu C Zhao W Han X Zhao H. Effects of extraction methods on the structure and functional properties of soluble dietary fiber from blue honeysuckle (*Lonicera caerulea* L.) berry. Food Chem. (2024) 431:137135. 10.1016/j.foodchem.2023.137135 37591145

[8] Siddiqui H Sultan Z Yousuf O Malik M Younis K. A review of the health benefits, functional properties, and ultrasound-assisted dietary fiber extraction. Bioact Carbohydr Diet Fibre. (2023) 30:100356. 10.1016/j.bcdf.2023.100356

[9] Miehle E Hass M Bader-Mittermaier S Eisner P. The role of hydration properties of soluble dietary fibers on glucose diffusion. Food Hydrocolloid. (2022) 131:107822. 10.1016/j.foodhyd.2022.107822

[10] Meng K Wang Y Liu F Zhan Q Zhao L. Effect of modifications on structure, physicochemical properties and lead ions adsorption behavior of dietary fiber of *Flammulina velutipes*. Food Chem. (2025) 464:141597. 10.1016/j.foodchem.2024.141597 39396472

[11] Zhao W Aiqi Ren A Shan S Li Z Su R Yang R. Inhibitory effects of soluble dietary fiber from foxtail millet on colorectal cancer by the restoration of gut microbiota. J Agri Food Chem. (2024) 72:12130–45. 10.1021/acs.jafc.4c00867 38748495

[12] Ding XM Zhang X Wei XY Wu RQ Gu Q Zhou T. Hypoglycemic and gut microbiota-modulating effects of pectin from *Citrus aurantium* “Changshanhuyou” residue in type 2 diabetes mellitus mice. J Agri Food Chem. (2025) 73:9088–102. 10.1021/acs.jafc.5c00547 40191895

[13] Torbica A Radosavljević M Belović M Djukić N Marković S. Overview of nature, frequency and technological role of dietary fibre from cereals and pseudocereals from grain to bread. Carbohyd Polym. (2022) 290:119470. 10.1016/j.carbpol.2022.119470 35550765

[14] Elleuch M Bedigian D Roiseux O Besbes S Blecker C Attia H. Dietary fibre and fibre-rich by-products of food processing: characterisation, technological functionality and commercial applications: a review. Food Chem. (2011) 124:411–21. 10.1016/j.foodchem.2010.06.077

[15] Dong R Liao W Xie J Chen Y Peng G Xie J. Enrichment of yogurt with carrot soluble dietary fiber prepared by three physical modified treatments: microstructure, rheology and storage stability. Innov Food Sci Emerg. (2022) 75:102901. 10.1016/j.ifset.2021.102901

[16] Kanwar P Yadav RB Yadav BS. Cross-linking, carboxymethylation and hydroxypropylation treatment to sorghum dietary fiber: effect on physicochemical, micro structural and thermal properties. J Cereal Sci. (2023) 233:123638. 10.1016/j.ijbiomac.2023.123638 36775223

[17] Zadeike D Vaitkeviciene R Degutyte R Bendoraitiene J Rukuiziene Z Cernauskas D. A comparative study on the structural and functional properties of water-soluble and alkali-soluble dietary fibers from rice bran after hot-water, ultrasound, hydrolysis by cellulase, and combined pre-treatments. Int J Food Sci Tech. (2021) 57:1137–49. 10.1111/ijfs.15480

[18] Xu B Zhang A Zheng Y Wang H Zheng X Jin Z. Influences of superfine-grinding and enzymolysis separately assisted with carboxymethylation and acetylation on the *in vitro* hypoglycemic and antioxidant activities of oil palm kernel expeller fibre. Food Chem. (2024) 449:139192. 10.1016/j.foodchem.2024.139192 38583404

[19] Huang F Tian Z Wang Y Ji X Wang D Fatehi P. Cellulose fiber drainage improvement via citric acid crosslinking. Int J Biol Macromol. (2024) 281:136338. 10.1016/j.ijbiomac.2024.136338 39374719

[20] Zheng YJ Tian HL Li Y Wang X Shi PQ. Effects of carboxymethylation, hydroxypropylation and dual enzyme hydrolysis combination with heating on physicochemical and functional properties and antioxidant activity of coconut cake dietary fibre. Food Chem. (2021) 336:127688. 10.1016/j.foodchem.2020.127688 32768904

[21] Wu Q Yao R Deng H Xiao B Ye Z Li Y. Synergistic interactions of citric acid grafted β-cyclodextrin and polyethyleneimine for improving interfacial properties of basalt fiber/epoxy composites. Compos Sci Technol. (2024) 251:110575. 10.1016/j.compscitech.2024.110575

[22] AOAC. Official Methods of Analysis. Washington, DC: Association of Official Analytical Chemists (2000).

[23] Chu J Zhao H Lu Z Lu F Bie X Zhang C. Improved physicochemical and functional properties of dietary fiber from millet bran fermented by *Bacillus natto*. Food Chem. (2019) 294:79–86. 10.1016/j.foodchem.2019.05.035 31126508

[24] Chandrasekara A Shahidi F. The content of insoluble bound phenolics in millets and their contribution to antioxidant capacity. J Agri Food Chem. (2010) 58:6706–14. 10.1021/jf100868b 20465288

[25] Berktas S Cam M. Effects of acid, alkaline and enzymatic extraction methods on functional, structural and antioxidant properties of dietary fiber fractions from quince (*Cydonia oblonga* Miller). Food Chem. (2025) 464:141596. 10.1016/j.foodchem.2024.141596 39413597

[26] Toma RB Leung HK. Determination of reducing sugars in French fried potatoes by 3,5-dinitrosalicylic acid. Food Chem. (1987) 23:29–33. 10.1016/0308-8146(87)90024-0

[27] Wang K Li M Han Q Fu R Ni Y. Inhibition of α-amylase activity by insoluble and soluble dietary fibers from kiwifruit (*Actinidia deliciosa*). Food Biosci. (2021) 42:101057. 10.1016/j.fbio.2021.101057

[28] Zhong K Jiang M Cao W Gao W Zheng H Lin H. Interaction between oyster peptides and anthocyanins: stability improvement, structure changes and α-amylase and α-glucosidase inhibition effect. LWT-Food Sci Technol. (2025) 221:117592. 10.1016/j.lwt.2025.11759

[29] Zhang S Dong Z Shi J Yang C Fang Y Chen G. Enzymatic hydrolysis of corn stover lignin by laccase, lignin peroxidase, and manganese peroxidase. Bioresource Technol. (2022) 361:127699. 10.1016/j.biortech.2022.127699 35905874

[30] Kim SR Park JY Park EY. Effect of ethanol, phytic acid and citric acid treatment on the physicochemical and heavy metal adsorption properties of corn starch. Food Chem. (2024) 431:137167. 10.1016/j.foodchem.2023.137167 37604005

[31] Jiao S Guo Q Ren W Zhou M Dai S Zhao Y. Production, structural and functional properties of dietary fiber from prosomillet bran obtained through Bifidobacterium fermentation. Food Chem. (2025) 475:143264. 10.1016/j.foodchem.2025.143264 39954644

[32] Tang C Wu L Zhang F Kan J Zheng J. Comparison of different extraction methods on the physicochemical, structural properties, and *in vitro* hypoglycemic activity of bamboo shoot dietary fibers. Food Chem. (2022) 386:132642. 10.1016/j.foodchem.2022.132642 35349899

[33] Wang Y Suo S Shang Y Pan D Jia L Lan J. Deciphering the effect of soluble dietary fiber from broccoli stem and leaf on metabolic syndrome and its hypoglycemic mechanism in diabetes mellitus. Food Biosci. (2025) 68:106546. 10.1016/j.fbio.2025.106546

[34] Dhital S Gidley MG Warren FJ. Inhibition of α-amylase activity by cellulose: kinetic analysis and nutritional implications. Carbohyd Polym. (2015)123:305–12. 10.1016/j.carbpol.2015.01.039 25843863

[35] Ahmed K Naymul K Mohammad R Bao T Yang L Wei C. Jujube fruit: a potential nutritious fruit for the development of functional food products. J Funct Foods. (2020) 75:104205. 10.1016/j.jff.2020.104205

[36] Bhatt S Gupta M. Dietary fiber from fruit waste as a potential source of metabolites in maintenance of gut milieu during ulcerative colitis: a comprehensive review. Food Res Inter. (2023) 164:112329. 10.1016/j.foodres.2022.112329 36737922

[37] Qin W Sun L Miao M Zhang G. Plant-sourced intrinsic dietary fiber: physical structure and health function. Trends Food Sci Technol. (2021) 118:341–55. 10.1016/j.tifs.2021.09.022

[38] Zheng YJ Li Y. Physicochemical and functional properties of coconut (*Cocos nucifera* L) cake dietary fibres: Effects of cellulase hydrolysis, acid treatment and particle size distribution. Food Chem. (2018) 257:135–42. 10.1016/j.foodchem.2018.03.012 29622189

